# ISCEV standard full-field ERG reference limits from 407 healthy subjects, derived from transference and validation of reference data between electrode types and centres

**DOI:** 10.1007/s10633-025-10009-2

**Published:** 2025-04-01

**Authors:** Rebecca A. Baker, Shaun M. Leo, William I. N. Clowes, Isabelle Chow, Xiaofan Jiang, Anne L. Georgiou, Antonio Calcagni, Christopher J. Hammond, Magella M. Neveu, Omar A. Mahroo, Anthony G. Robson

**Affiliations:** 1 Moorfields Eye Hospital NHS Foundation Trust, 162 City Road, London, EC1V 2PD UK; 2 UCL Institute of Ophthalmology, London, UK; 3 St Thomas’ Hospital, London, UK

**Keywords:** Reference range, Normative data, Silver thread electrode, Electroretinogram, Full-field ERG, Human, Clinical electrophysiology of vision

## Abstract

**Purpose:**

To establish age-adjusted reference intervals for the ISCEV standard full-field electroretinogram (ERG) recorded with silver thread electrodes in the lower fornix, based on a combined reference sample involving recordings from reference subjects and transference of data between two centres and two types of electrode.

**Methods:**

Silver thread lower fornix ERG reference data from two centres underwent verification for inclusion in the reference sample (n = 251). Comparison analysis was performed to determine whether gold foil reference data could be included in the silver thread reference range, directly or with adjustment. Reference subjects and patients underwent ERG testing with both silver thread and gold foil electrodes (n = 53) and skin electrodes (n = 41). A linear model, fitted to the electrode comparison data, was used to transform gold foil ERG reference data for inclusion in the reference sample (n = 156). The combined sample of 407 reference individuals was used to derive age-adjusted reference limits for the main DA 0.01, DA 3, DA 10, LA 30 Hz and LA 3 ERG components.

**Results:**

Silver thread ERG reference data was sufficiently similar across two centres to justify combination into a single reference sample. Peak times for gold foil and silver thread ERGs were closely comparable (r^2^ 0.75–0.98, Bland–Altman bias ≤ 1.6 ms for all ERG components), with LA 30 Hz peak time showing the highest agreement (bias: − 0.2 ms, 95% limits of agreement (LOA): − 1.1 to 0.7 ms, ‘silver thread—gold foil’). There was a clinically significant amplitude difference between electrode types: silver thread ERGs were 55–65% of the amplitude of gold foil ERGs (LOA ranged from 29 to 90%) and skin ERGs were 35–38% of the amplitude of silver thread ERGs (LOA ranged from 18 to 54%). Pooled reference data formed a sufficient sample covering 8 decades, from which age-adjusted parametric and nonparametric reference limits were calculated with reference to current guidelines.

**Conclusions:**

ISCEV standard silver thread ERG data were consistent across the two centres, allowing transference of reference data. Reference data recorded with gold foil electrodes can be transformed for inclusion in a silver thread ERG reference range. The study highlights methods of pooling multiple sources of reference data into a larger, more robust sample, pertinent to standardization, clinical management, and multi-centre studies. These reference data could be adopted by other centres or combined with other datasets, following suitable verification.

**Supplementary Information:**

The online version contains supplementary material available at 10.1007/s10633-025-10009-2.

## Introduction

The full-field electroretinogram (ERG) measures the retinal response to diffuse flashes of light and is used routinely in the diagnosis, monitoring, and management of retinal disease. The International Society for the Clinical Electrophysiology of Vision (ISCEV) Standard for clinical full-field ERG describes a minimum ERG protocol for the assessment of retinal function under dark-adapted (DA) and light-adapted (LA) conditions [1]. The use of varying flash strengths and frequencies evokes global retinal responses which can be used to characterize outer and inner retinal rod and cone system function, with wide clinical applicability [2]. The ISCEV Standards serve not only to generate high quality, clinically relevant data but also to promote inter-centre consistency, with centres that conform to the standards producing highly consistent stimuli and ERG responses [3, 4].

Interpretation of the ERG typically requires comparison against a sample of individuals without retinal dysfunction who are demographically matched to the patient population, known as the reference sample [1]. The ISCEV Standard notes the need for a sufficient sample to allow nonparametric calculation of the 2.5th and 97.5th percentile [1]. Published methods suggest a minimum of 120 individuals for each clinically relevant partition, with precision defined according to 90% confidence limits [5]. Age-related changes in ERGs must also be considered, as responses mature from early infancy and may decrease in amplitude and increase in peak time over adult lifespan [6–9]. The collection of laboratory-specific ERG reference data is optimal, since local testing methods affect response characteristics. Of particular importance is the type of active electrode, which significantly alters ERG amplitude [10]. Electrode positioning also affects ERG amplitude: silver thread electrodes (STEs) placed along the lower lid margin yield ERGs that are 12–19% larger than ERGs with silver thread in the fornix position [11]. Although seen as the ‘gold standard’, the collection of laboratory-specific reference data is highly time-consuming as it involves testing large numbers of reference subjects, and new reference data may be needed if test protocols are changed [5].

Methods have been developed to enable transference of reference data between laboratories and electrode types, including the use of legacy data following a change in methods or the adoption of published reference ranges, providing that data is suitably verified [1, 5, 12]. Although first described for use in clinical laboratory medicine, the relevance of these methods to visual electrophysiology has been highlighted [5], made possible by the standardization of test stimuli and recording protocols [1] as demonstrated across centres [3, 4]. Such methods may improve access to high quality reference data, which can be adopted locally following verification with as few as 20 local reference subjects [5]. This study aimed to establish age-adjusted reference intervals for ISCEV standard ERGs recorded with silver thread corneal electrodes positioned in the lower fornix, using published methods to transfer and verify reference data from different laboratories and electrode types.

## Methods

The study took place across two centres: Moorfields Eye Hospital (MEH) and St Thomas’ Hospital (STH), in London, UK. Participants underwent full-field ERG testing according to the ISCEV Standard [1], with ERG stimuli delivered via a ganzfeld bowl (Colordome, Diagnosys). Test equipment and stimulus settings were consistent across centres, and consistency of responses was confirmed in 6 participants who underwent ERG testing with silver thread electrodes at both centres. Full-field ERGs incorporated the ISCEV Standard and included the DA 0.01, DA 3, DA 10 and LA 30 Hz and LA 3 ERGs. DA responses were recorded after 20 min of dark adaptation and LA ERGs after 10 min of light adaptation to a background luminance of 30 cd·m^−2^. Pupils were dilated with mydriatic drops (tropicamide 1% and, if not contraindicated, phenylephrine hydrochloride 2.5%). Detailed parameters are available as supplementary information (Online Resource 1).

**Fig. 1:**
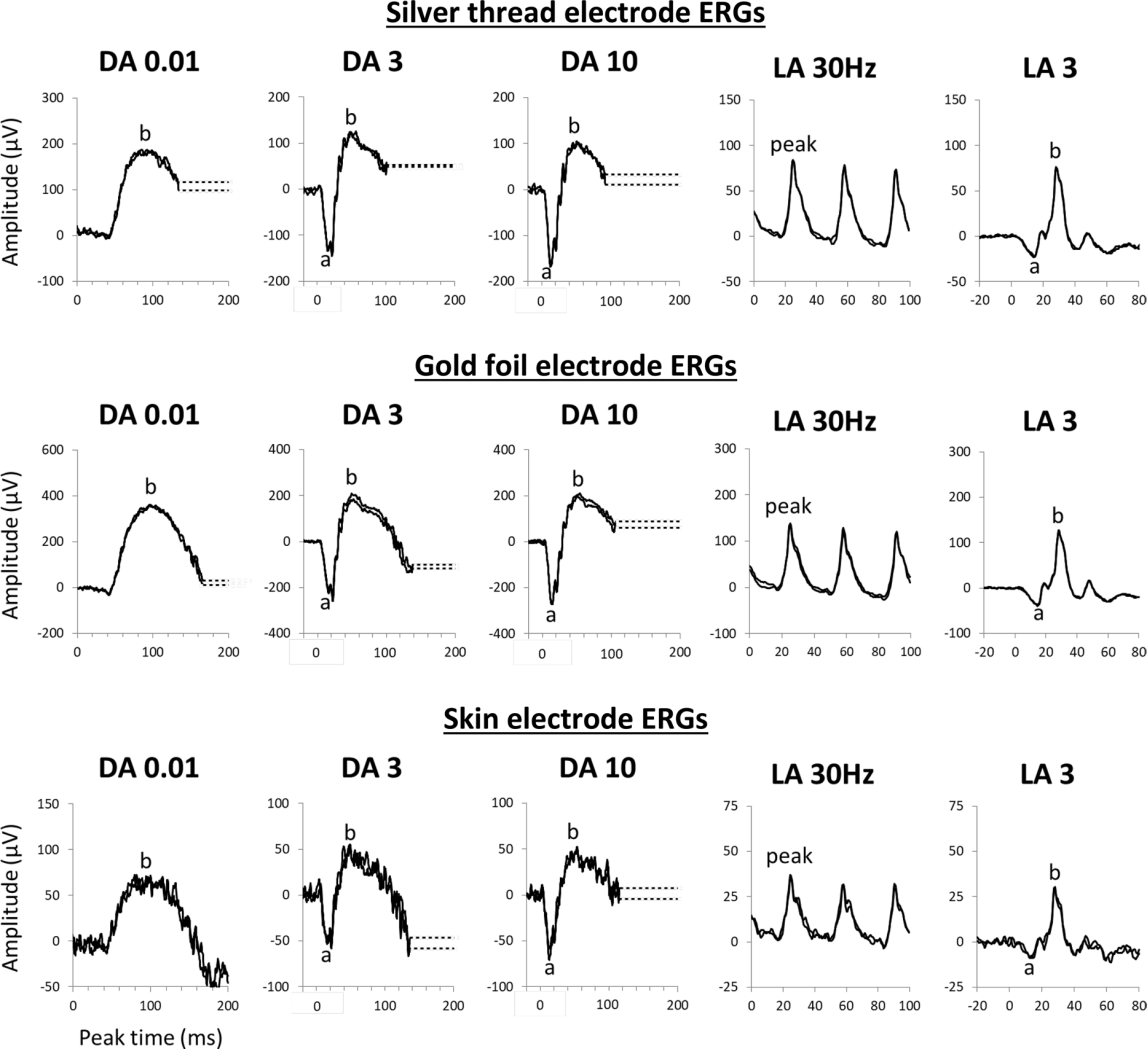
Representative ISCEV full-field ERG waveforms recorded from a 42-year-old reference subject. Recordings were performed with three electrode types simultaneously using a silver thread electrode in the lower fornix position of the left eye, a gold foil electrode in the right eye, and skin electrodes for both eyes (skin electrode responses were checked for consistency between eyes and are shown from one eye only). Note the differing y-axis scale. Dashed lines replace blink artefact occurring after the ERG b-wave. When a-waves had a bifid appearance, as commonly seen in the DA 3 ERG, the earliest negative component was measured

The reference range was composed of three distinct datasets: (1) silver thread ERGs from volunteer reference subjects at MEH (2) gold foil ERGs from volunteer reference subjects and patients without eye disease at MEH, adjusted according to a comparison analysis (see *Comparison Analysis*), and (3) silver thread ERGs from volunteer reference subjects at STH. These are described in more detail below (see *Datasets*). Silver thread ERGs were recorded with the ‘DTL Plus Electrode’ from Diagnosys, or the ‘Sterile ERG Thread Electrode’ from Spes Medica, placed in the lower conjunctival fornix (‘fornix position’). Both silver thread electrodes were composed of silver-nylon threads secured at either side of the eye by adhesive pads, and consistency of ERG responses between the two silver thread electrodes was confirmed in four normal subjects (see Online Resource 2). Gold foil ERGs were recorded with gold foil electrodes (GFEs, CH Electronics) centred beneath the pupil at the corneoscleral junction. Some participants had ERGs recorded with lower eyelid skin electrodes (Ambu Neuroline), which were analysed for comparison but not included in the reference range. Responses were recorded using the Espion E3 system (Diagnosys). In all datasets, the ground and reference electrodes used were skin-surface electrodes and were placed at the forehead and ipsilateral outer canthus respectively. At least three consistent traces were recorded at each step, with component amplitudes and peak times measured as in Fig. 1.

### Datasets

#### Dataset 1a and 1b: MEH reference subjects tested with silver thread electrodes

Silver thread ERGs were examined retrospectively from a convenience sample of volunteers who had given informed consent to be tested as reference subjects. Values were excluded if the participant reported retinal or optic nerve disease in either eye (n = 2), or if responses could not be measured reliably due to technical factors or artefacts (n = 4–12, different for each ERG component). After exclusions, twelve participants who had undergone binocular silver thread ERG testing were included in the reference range (left eye values) (Dataset 1a). A further 41 participants had undergone simultaneous testing with silver thread (left eye), gold foil (right eye), and skin electrodes (binocular) such that each eye had two active electrodes each referred to the ipsilateral outer canthus reference electrode (Dataset 1b). This allowed comparison of gold foil and silver thread ERGs recorded simultaneously in the same subject, with skin electrodes confirming inter-ocular symmetry (this electrode montage was validated prior to use, see Online Resource 2). For these participants (Dataset 1b) the left eye silver thread ERG values were included in the reference range (n = 37 after exclusions due to technical issues such as movement of electrode to lower lid position), while gold foil and skin ERGs were analysed for comparison (see *Comparison Analysis*). One additional reference subject was tested with silver thread and gold foil electrodes (without skin electrodes) according to the protocol described below (Dataset 4).

#### Dataset 2: MEH reference subjects tested with gold foil electrodes

ISCEV Standard reference ERG data obtained with gold foil recording electrodes was extracted retrospectively from recordings at MEH between August 2011 and November 2020. All patients and volunteers had given their informed consent for testing (for paediatric patients, parental consent was obtained). The sample included healthy volunteers (n = 20) and a cohort of ophthalmologically normal patients. The patients included cases confirmed to have non-organic visual loss (n = 81) or medically unexplained visual symptoms with normal ocular exam (n = 30), cases of congenital sensorineural hearing loss subsequently found to be without retinopathy (n = 8), individuals that were tested due to a family history of retinal dystrophy that were subsequently considered to be unaffected clinically (n = 4), patients with congenital hypertrophy of the retinal pigment epithelium (n = 1), and patients who were diagnosed with non-retinal pathology including migraine (n = 3), visual snow/pixelated vision (n = 6) or strabismus and ocular motility disorders (n = 3). Gold foil ERG amplitudes were transformed to account for the smaller amplitude of silver thread recordings, using the linear model derived from the comparison analysis (see *Comparison Analysis*). One eye per patient was included: in most instances this was the right eye (n = 145), although if right eye values were unavailable or affected by artefact then left eye values were used (n = 11). For validation, the number of Dataset 1 (silver thread) values falling outside of the 95% nonparametric reference interval derived from Dataset 2 (gold foil ERGs, following adjustment to account for amplitude differences between electrode types) was counted to confirm that a suitably low proportion (< 10% [12]) fell outside the reference limits.

#### Dataset 3: STH reference subjects tested with silver thread electrodes

A sample of twins from the TwinsUK Cohort at STH was verified for inclusion in the reference range, most of which have been described previously [6]. Eight participants with ocular disease were excluded (4 due to glaucoma, 3 due to age-related macular degeneration, and 1 with past retinal detachment). Testing was with silver thread electrodes in the fornix position (DTL-plus electrode; Unimed Electrode Supplies). One eye per subject was included: in most instances this was the right eye (n = 192), although if right eye values were unavailable or affected by artefact then left eye values were used (n = 9). For verification, the number of Dataset 1 (MEH) values falling outside of the 95% nonparametric reference interval derived from Dataset 3 (STH) was counted to confirm that a suitably low proportion (< 10% [12]) fell outside the reference limits.

#### Dataset 4: patients tested with gold foil and silver thread electrodes

Clinical data collected between February and May 2023 were examined retrospectively from fourteen patients who had undergone ERG testing with gold foil and silver thread electrodes in the same session, to be used for electrode comparison (not included in reference range: see *Comparison Analysis*). This included patients with retinal dysfunction, although undetectable responses were excluded from the analysis due to potential to excessively influence the linear model. All patients had given their informed consent for testing. The test order was as follows: after 20 min dark-adaptation the DA ERGs were recorded with silver thread electrodes followed immediately by DA ERGs with gold foil electrodes, with the electrodes changed under dim red-light conditions. At the end of DA ERG testing patients were then light adapted for 10 min and underwent LA ERG testing with gold foils and then silver threads.

### Comparison analysis

The aim of the comparison analysis was to derive a model to describe the relationship between ERGs recorded with silver thread and gold foil electrodes. This model was used to adjust gold foil ERG reference data for inclusion in the reference range. To ensure the model covered a range of amplitudes and peak times, both volunteers (n = 42 from dataset 1) and patients (n = 14 from dataset 4) were included [5].

Regression analysis was based on published methods [5, 12, 13]. To compare electrode types, gold foil ERG amplitudes and peak times were plotted against silver thread ERG amplitudes and peak times for each component. A linear regression was performed, along with quantile–quantile (Q–Q), residual, and Bland–Altman plots. Components which were highly correlated and linearly related (r^2^ ≥ 0.7) were transferred between electrodes using the regression slope and y-intercept values as correction factors (for a specific example see Table 2 legend). Where r^2^ ≥ 0.95, the slope and y-intercept of a simple linear regression was applied to gold foil reference data. Where r^2^ was < 0.95, the slope and y-intercept of a Deming regression was applied to gold foil reference data. The Bland–Altman method was used to compare skin ERGs with gold foil and silver thread ERGs. For ease of interpretation, Bland–Altman bias was expressed as a ratio for amplitudes and an absolute difference in milliseconds for peak times.

### Calculation of reference limits

Two methods were used for calculation of reference intervals. Analysis was with Microsoft Excel using macroinstructions ‘Reference Value Adviser V2.1’, the details of which are published elsewhere [14].

- Nonparametric method:
  The sample was divided into three age groups of similar sample size: ≤ 35 years, 36–59 years, ≥ 60 years, each containing > 120 reference individuals. The age range groups were partly based on data availability but also on the need to maintain a minimum and approximately equal sample in excess of 120 data points within each partition, thereby allowing precise computation of the nonparametric 95% reference limits and 90% confidence interval for each age group and ERG parameter. An exception was made for ERG parameters that did not change with age: in such instances, division by age was not justified and nonparametric reference limits were calculated on the whole sample. To account for the heritability of ERG characteristics [6] and the paired nature of data in the twins cohort (dataset 3), nonparametric reference limits were additionally recalculated with one twin from each pair excluded.
- Parametric method following Log_10_ or Box-Cox transformation:
  The combined reference sample was plotted against age for each ERG component. As the distribution of values at each age had a positive skew, Log_10_ (for lognormal distributions) or Box-Cox (for distributions that were neither Gaussian nor lognormal) transformation was performed to achieve an approximately Gaussian distribution. Simple linear regression and parametric upper and lower 95% reference limits (with 90% CI) were calculated on the transformed values, then back transformed to obtain the fitted values plotted graphically against age (details of the method used have been published previously [14]). Weighted residuals were confirmed to be evenly distributed around 0 with no discrete groups or trends.

## Results

### Participants

A total of 407 participants were included in the combined reference sample. The sample was 83.5% female and mostly white European. Age ranged from 7 to 86 years (mean age 47 years). Participants in the St Thomas’ Hospital sample (dataset 3) were significantly older than the other two groups (*p* < 0.0001, Kruskal–Wallis test with Dunn’s multiple comparisons) and had a higher proportion of females (*p* < 0.0001, Chi-square test), with no significant age or sex difference between the other two samples. The combined reference sample comprised of 271 volunteers and 136 patients, the patients having been confirmed free of retinal/optic nerve disease. Characteristics and demographics for each dataset are summarized in Table 1.

**Table 1:** Sample characteristics for each dataset

Dataset	n =	Active electrode	Eye included	Mean age (± SD)	Sample characteristics	% female
				*Median*		
1a	50	Silver thread only (n = 12)	Left	37.2 (± 11.7)	Reference individuals without retinal or optic nerve disease	68.6
1b		Silver thread, gold foil, and skin (n = 37)		*37.1*		
		Silver thread and gold foil (n = 1)				
2	156	Gold foil	145 right	30.3 (± 17.4)	20 reference individuals without retinal or optic nerve disease	74.4
			11 left	*25.8*	136 patients without retinal or optic nerve disease	
3	201	Silver thread	192 right	62.0 (± 11.2)	Reference individuals without retinal or optic nerve disease:	94.0
			9 left	*64.3*	96 twin pairs (monozygotic and dizygotic)	
					9 individual twins (pair excluded)	
4	14	Silver thread and gold foil	6 right	58.1 (± 15.3)	Patients with or without retinal or optic nerve disease	57.1
			8 left	*60.5*		
Total in reference range 1, 2 & 3)	407	Silver thread (n = 254)	337 right	46.8 (± 20.6)	271 reference individuals	83.5
		Gold foil (n = 156)	70 left	*52.3*	136 patients without retinal or optic nerve disease	

*SD* standard deviation

### Comparison of gold foil, silver thread, and skin electrode ERGs

**Fig. 2:**
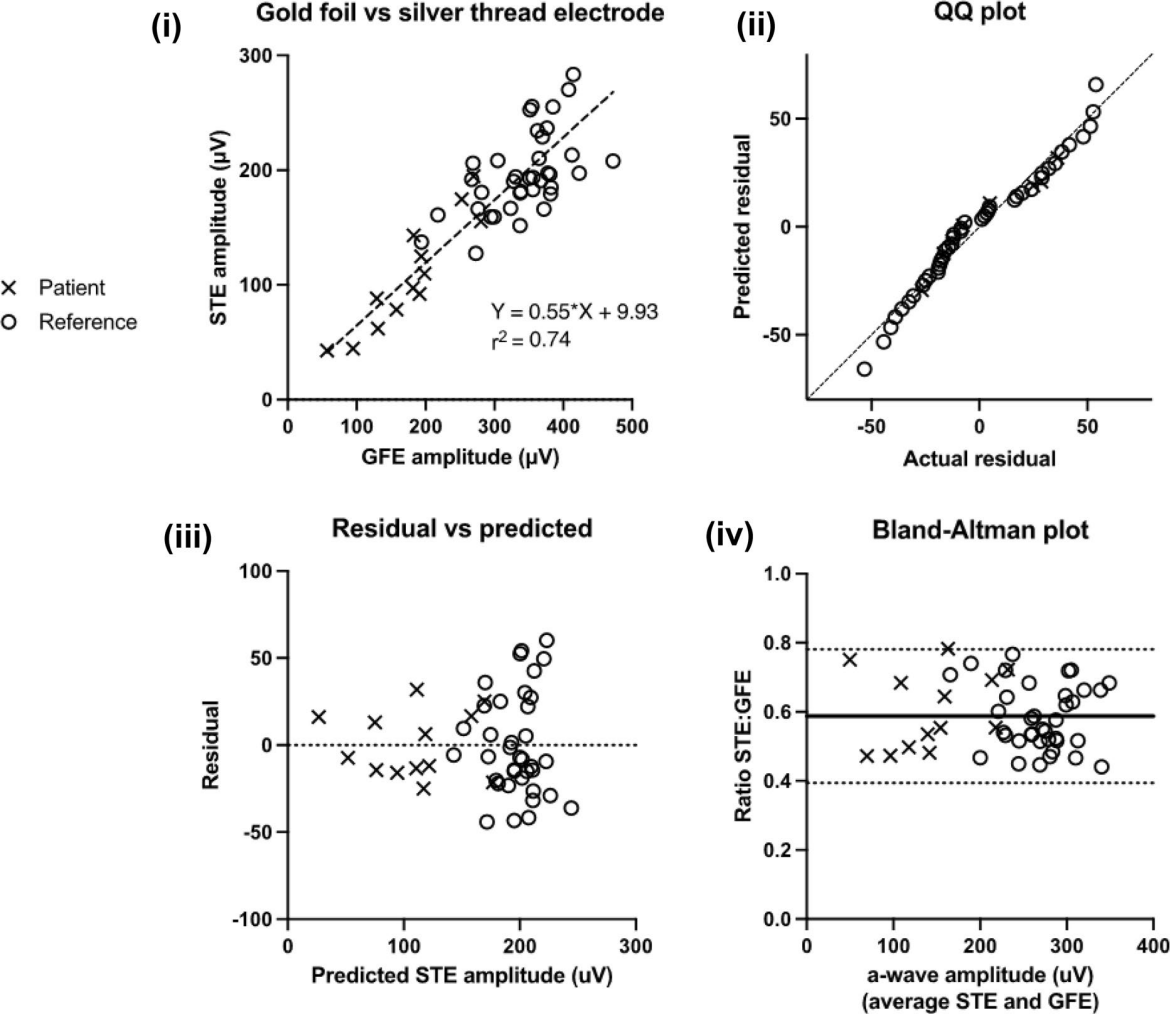
Comparison analysis of DA 10 a-wave amplitude recorded with gold foil and silver thread electrodes. (i) DA 10 a-wave peak time recorded with gold foil and silver thread electrodes in reference subjects and patients. Dashed line shows a Deming’s regression. (ii) QQ plot illustrates the normality of errors. (iii) Residual plot illustrates the spread of data in relation to the predicted value (see text). (iv) Bland–Altman plot illustrates the bias (bold line) and 95% limits of agreement (dotted lines) of the DA 10 a-wave amplitude when recorded with silver threads compared with gold foils

**Fig. 3:**
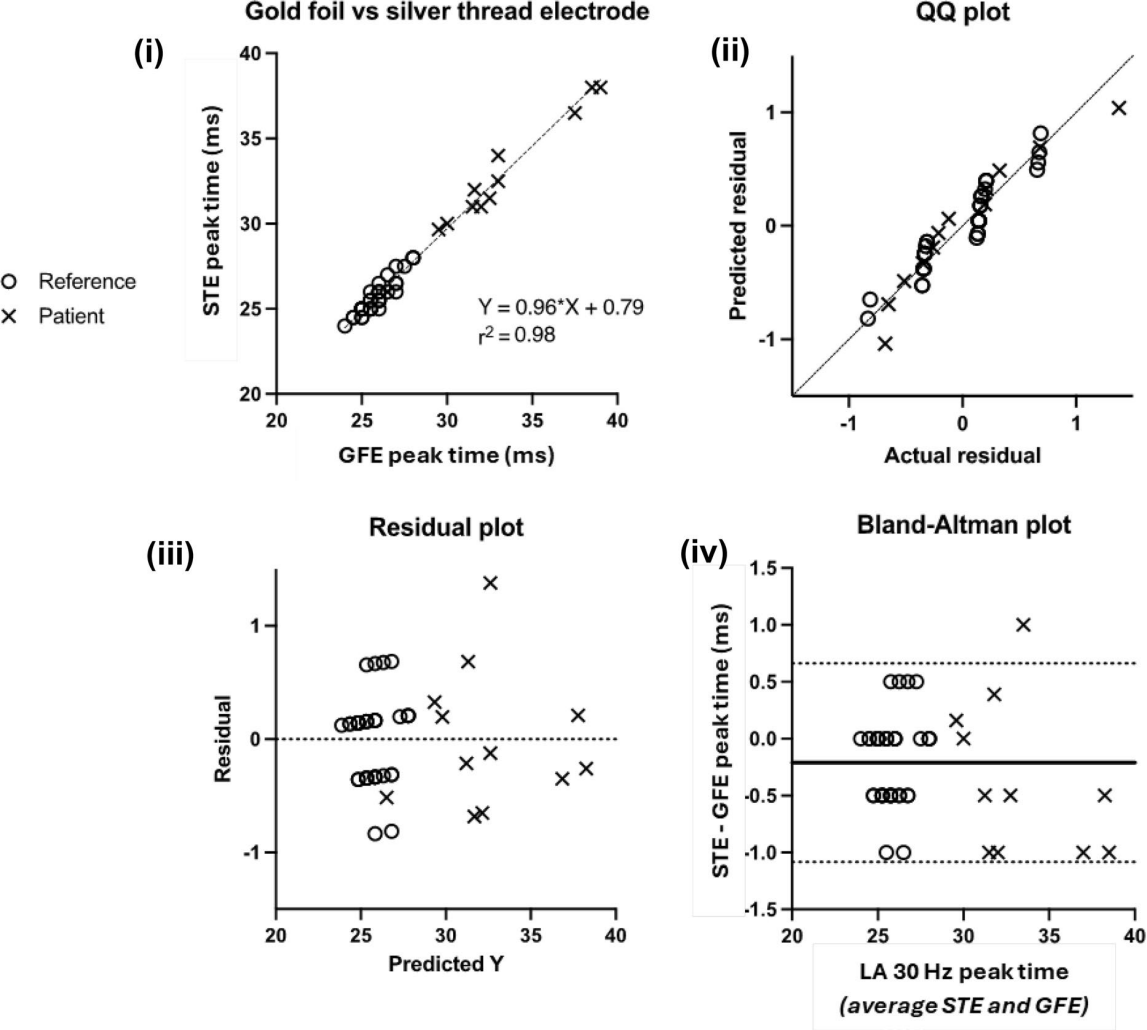
Comparison analysis of LA 30 Hz peak time recorded with gold foil and silver thread electrodes. (i) LA 30 Hz peak time recorded with gold foil and silver thread electrodes in reference subjects and patients. Dashed line shows a simple linear regression. (ii) QQ plot illustrates the normality of errors. (iii) Residual plot illustrates the spread of data in relation to the predicted value (see text). (iv) Bland–Altman plot illustrates the bias (bold line) and 95% limits of agreement (dotted lines) of the LA 30 Hz ERG peak time when recorded with silver threads compared with gold foils

Comparison analysis of ERGs recorded with gold foil and silver thread electrodes was undertaken on 52 participants (datasets 1b and 4). An additional 4 subjects excluded due to technical issues with the silver thread electrodes were included in the gold foil/skin electrode comparison analysis. Illustrative scatter plots are shown in Figs. 2 and 3. ERGs recorded with gold foil and silver thread electrodes correlated. LA 30 Hz peak time showed the highest correlation (r^2^ = 0.98), while LA 3 a-wave amplitude showed the lowest correlation between electrode types (r^2^ = 0.68). LA 30 Hz and DA 3 a-wave peak times were the only components for which r^2^ ≥ 0.95 and were analysed with simple linear regression. The rest of the comparison data was modelled using Deming regression (r^2^ = 0.68–0.94). LA 3 a-wave amplitude was the only ERG component not attaining the r^2^ value of ≥ 0.7, which was set as the validation threshold as per published methods [5]. Given that the ERG components are interdependent and the successful validation of all other ERG components, this borderline case was considered acceptable and LA 3 gold foil amplitude data was included in the reference range following conversion.

Gold foil and silver thread ERG amplitudes were linearly related (Fig. 2) but silver thread ERGs were of significantly lower amplitude compared with gold foil ERGs (slope of the linear regression < 1 for all ERG component amplitudes, see Table 2). Residuals plots revealed some evidence of heteroscedasticity, with higher errors for larger amplitudes, although as this was minimal the linear model was judged to be a suitable fit for the data. Bland–Altman plots for amplitude revealed a difference between ERGs recorded with silver thread and gold foil electrodes, with silver thread ERGs being 55–65% (mean 60%; SD 3.5%) the amplitude of gold foil ERGs (LOA ranged from 29 to 90%). The ERG component-specific slope and intercept of the linear model was applied to gold foil amplitudes (Dataset 2) for inclusion in the combined reference range.

**Table 2:** Linear regression and Bland–Altman analysis of ERG components recorded with gold foil and silver thread electrodes

Amplitude	n =	r^2^	Slope	Intercept	Bias (STE:GFE)	Result
			*95% CI*	*95% CI*	*95% LOA*	
*DA 0.01 b-wave*	46	0.73	0.46^†^	24.38^†^	0.55	Accept after adjustment
			*0.38–0.54*	− *0.04 to 48.81*	*0.33 to 0.77*	
*DA 3 a-wave*	49	0.79	0.56^†^	8.24^†^	0.61	Accept after adjustment
			*0.46 to 0.63*	− *3.80 to 31.38*	*0.41 to 0.82*	
*DA 3 b-wave*	47	0.73	0.48^†^	36.75^†^	0.57	Accept after adjustment
			*0.40 to 0.56*	*5.90 to 67.60*	*0.38 to 0.76*	
*DA 10 a-wave*	50	0.74	0.55^†^	9.93^†^	0.59	Accept after adjustment
			*0.45 to 0.64*	− *15.16 to 35.02*	*0.39 to 0.78*	
*DA 10 b-wave*	49	0.72	0.48^†^	37.92^†^	0.57	Accept after adjustment
			*0.39 to 0.57*	− *1.18 to 77.02*	*0.37 to 0.78*	
*LA 30 Hz peak*	52	0.85	0.73^†^	− 9.54^†^	0.65	Accept after adjustment
			*0.63 to 0.83*	− *20.41 to 1.32*	*0.42 to 0.89*	
*LA 3 a-wave*	52	0.68	0.55^†^	1.15^†^	0.59	Borderline
			*0.42 to 0.69*	− *4.03 to 5.82*	*0.29 to 0.90*	
*LA 3 b-wave*	52	0.81	0.65^†^	− 3.00^†^	0.64	Accept after adjustment
			*0.53 to 0.76*	− *17.91 to 11.91*	*0.41 to 0.87*	

Peak time	n =	r^2^	Slope	Intercept	Bias (ms) (STE–GFE)	Result
			*95% CI*	*95% CI*	*95% CI*	
*DA 0.01 b-wave*	46	0.87	1.05^†^	− 5.91^†^	− 1.6	Accept
			*0.91 to 1.18*	− *17.6 to 5.82*	− *9.3 to 6.2*	
*DA 3 a-wave*	49	0.95	1.03*	− 0.81*	− 0.3	Accept
			*0.96 to 1.10*	− *1.97 to 0.35*	− *1.3 to 0.8*	
*DA 3 b-wave*	47	0.89	0.94^†^	1.63^†^	− 1.5	Accept
			*0.85 to 1.04*	− *3.46 to 6.73*	− *4.7 to 1.7*	
*DA 10 a-wave*	50	0.85	1.12^†^	− 2.12^†^	− 0.5	Accept
			*0.95 to 1.28*	− *4.34 to 0.09*	− *2.0 to 1.0*	
*DA 10 b-wave*	49	0.89	0.94^†^	1.95^†^	− 1.1	Accept
			*0.80 to 1.09*	− *5.98 to 9.88*	− *4.8 to 2.6*	
*LA 30 Hz peak*	52	0.98	0.96*	0.79*	− 0.2	Accept
			*0.93 to 1.0*	− *0.15 to 1.7*	− *1.1 to 0.7*	
*LA 3 a-wave*	52	0.75	1.05^†^	− 0.89^†^	− 0.1	Accept
			*0.83 to 1.28*	− *4.19 to 2.41*	− *1.1 to 0.9*	
*LA 3 b-wave*	52	0.94	0.98^†^	0.21^†^	− 0.4	Accept
			*0.86 to 1.11*	− *3.43 to 3.85*	− *1.7 to 0.9*	

^*^Simple linear regression. ^*†*^*Deming regression. Numbers in italics show the 95% confidence interval (CI) or limits of agreement (LOA). Sample size varies between steps due to exclusions for technical reasons*/*artefacts*. For ERG component amplitudes, the slope and intercept of the regression line were applied as correction factors to gold foil reference values for inclusion in the silver thread reference range. For ERG component peak times, the slope of the regression line was considered close enough to 1 and intercept close enough to 0 to justify direct transfer without adjustment (also confirmed by the small/insignificant bias). For example, a reference subject with an LA 30 Hz flicker response at 27 ms and 100 µV recorded with gold foil electrodes would be included in the silver thread reference range with a peak time of 27 ms and an amplitude of 63.46 µV (= 100 µV * 0.73–9.54)

For peak time, there was no clinically significant difference between silver thread and gold foil ERGs. The slope of the linear regression was near 1 (mean = 1.0; SD = 0.06) and Y intercept near 0 for all ERG component peak times (see Table 2), and Q-Q and residuals plots supported a linear trend (see Fig. 3). Peak times were, on average, no more than 0.5 ms earlier in silver threads versus gold foils for all photopic ERG peak times and scotopic ERG a-wave peak times (Bland–Altman LOA [STE-GFE] ranged from − 2.0 to + 1.0 ms), with LA 30 Hz peak time being the most consistent component (LOA [STE-GFE] = − 1.1 to + 0.7 ms). Limits of agreement for scotopic ERG b-wave peak times were slightly wider but still highly consistent, with a bias of ≤ 1.6 ms in silver threads versus gold foil electrodes (with silver threads having marginally but not significantly shorter peak times). As such, gold foil reference data for peak time (Dataset 2) were included in the reference range without any transformation.

ERGs with skin electrodes were on average 35–38% the amplitude of silver thread ERGs (LOA ranged from 18 to 54%, Online Resource 3) and 19–24% the amplitude of gold foil ERGs (LOA ranged from 10 to 34%, Online Resource 4). Peak times were highly consistent between skin and silver thread/gold foil electrodes, with a bias of 0.1 to 2.9 ms (‘STE’ - ‘skin’) for silver thread and 3.8 to − 0.9 ms (‘GFE’ - ‘skin’) for gold foil electrodes. Skin ERG amplitudes showed no interocular asymmetry (bias [RE:LE] ranged from 0.99 to 1.04, Online Resource 5), and differed by no more than a third in 95% of cases with the exception of LA 3 a-wave amplitude which had wider limits of agreement (bias [RE:LE] = 1.04, LOA = 0.60–1.48). Interocular bias was less than 1 ms for all skin ERG peak times.

### Validation

Datasets 2 and 3 were validated against silver thread ERGs from reference subjects in Dataset 1. Since participants in Datasets 1 and 2 were of comparable age, the 95% nonparametric reference interval was derived from the whole Dataset 2 sample (all ages). A suitably small proportion of values from Dataset 1 fell outside the nonparametric reference limits for Dataset 2 (< 10% for 7/8 component amplitudes and 7/8 component timings, mean: 5.5% for component amplitudes and 3.1% for component timings: see Table 3). Since participants in Dataset 3 were significantly older than in Dataset 1, only participants aged 30–60 years were compared. A suitably small proportion of values from Dataset 1 fell outside the nonparametric reference limits for Dataset 3 (< 10% for 6/8 component amplitudes and 6/8 component timings, mean: 5.3% for component amplitudes and 6.8% for component timings: see Table 3). Repeat ERGs recorded at both centres (MEH and STH) in 6 healthy volunteers (included in Dataset 1a) showed low inter-centre variability: ISCEV ERG component amplitudes differed by a mean of 7% (range 0–23% excluding a single DA 0.01 outlier) and peak times differed by a mean of 4% (range 0–13% excluding a single DA 0.01 outlier) between centres. The datasets were judged to be sufficiently similar to justify combination into a single combined reference sample.

**Table 3:** Number of Dataset 1 values falling outside nonparametric reference limits constructed from Dataset 2 and Dataset 3

	Dataset 1 versus 2 (All ages)		Dataset 1 versus 3 (Age 30–60 only)	
	Amplitude	Peak time	Amplitude	Peak time
*DA 0.01 b-wave*	3/45 (6.7%)	2/45 (4.4%)	0/28 (0.0%)	3/28 (10.7%)
*DA 3 a-wave*	4/47 (8.5%)	1/47 (2.1%)	0/29 (0.0%)	2/29 (6.9%)
*DA 3 b-wave*	0/47 (0.0%)	1/47 (2.1%)	2/29 (6.9%)	1/29 (3.4%)
*DA 10 a-wave*	5/48 (10.4%)	1/48 (2.1%)	1/29 (3.4%)	1/29 (3.4%)
*DA 10 b-wave*	2/48 (4.2%)	5/48 (10.4%)	0/29 (0.0%)	1/29 (3.4%)
*LA 30 Hz peak*	3/49 (6.1%)	1/49 (2.0%)	5/31 (16.1%)	1/31 (3.2%)
*LA 3 a-wave*	3/49 (6.1%)	0/49 (0.0%)	2/31 (6.5%)	1/31 (3.2%)
*LA 3 b-wave*	1/49 (2.0%)	1/49 (2.0%)	6/31 (19.4%)	6/31 (19.4%)

The number of Dataset 1 values falling outside the reference interval is shown as a proportion of the total Dataset 1 sample, with brackets expressing this proportion as a percentage. Dataset 1 sample size varies between steps due to exclusions for technical reasons/artefacts

## Reference limits

The age-adjusted reference limits calculated according to nonparametric methods across three age groups are shown in Tables 4 and 5. The nonparametric reference interval for DA 10 ERG b-wave timing was calculated without partition by age (no significant difference between groups); all other nonparametric limits were calculated in three age subgroups. Partition by sex was not possible due to the small percentage of males in the sample (16.5%). The Clinical and Laboratory Standards Institute (CLSI) guidelines recommend that 90% CIs should be ≤ 20% of the total reference interval [12]. For ERG component timings, the 90% CI of the upper reference limits ranged from 9 to 50% of the total reference interval (only 7 of 22 computed upper limits were ≤ 20%). For ERG component amplitudes, the 90% CI of the lower reference limits ranged from 7 to 24% and was ≤ 20% for all but one parameter (LA 3 ERG a-waves in ages 36–59: 24%). Exclusion of paired twin data from the sample made little difference to the estimated reference limits (Online Resource 6).

**Table 4:** Age-specific nonparametric reference limits for timing of ISCEV-standard full-field ERGs with silver thread electrodes in the fornix position

Age	Nonparametric reference limits for ERG component timing (ms)		
	*90% CI of reference limits (CI as proportion of total reference interval)*		
	≤ 35	36–59	≥ 60
*DA 0.01*			
*b wave*	72–99.5 (n = 126)	77.5–107.5 (n = 130)	78–115 (n = 123)
	*66.5–75.5 (0.32) and 95–106 (0.40)*	*73–80 (0.23) and 104–118.5 (0.48)*	*77.5–86.5 (0.30) and 112.5–116 (0.09)*
*DA 3*			
*a wave*	13.5–16 (n = 130)	14.5–17 (n = 138)	14.5–18 (n = 130)
	*13–14 (0.42) and 16–17 (0.42)*	*14.5–14.5 (0.00) and 17–17.5 (0.20)*	*14–15 (0.30) and 17.5–18.5 (0.30)*
*b wave*	45.5–58.5 (n = 130)	46.5–59.5 (n = 138)	46.5–58 (n = 130)
	*42.5–47 (0.35) and 57.5–60 (0.19)*	*45–47 (0.16) and 56.5–59.5 (0.24)*	*45.5–47.5 (0.18) and 57–60 (0.26)*
*DA 10*			
*a wave*	10–14.5 (n = 134)	11–15 (n = 136)	11.5–15.5 (n = 131)
	*10–10.5 (0.12) and 13.5–14.5 (0.23)*	*10.5–11 (0.13) and 14.5–16.5 (0.50)*	*11–11.5 (0.13) and 15.5–16.5 (0.25)*
*b wave*	47–59.5 (n = 392)	*b-wave: see* ≤ *35 years*	*b-wave: see* ≤ *35 years*
	*46–47.5 (0.11) and 58.5–60 (0.13)*		
*LA 30 Hz*			
*peak*	24–28 (n = 121)	24–29.5 (n = 137)	24.5–30.5 (n = 127)
	*24–24.5 (0.13) and 27–28 (0.25)*	*24–24.5 (0.09) and 28–30.5 (0.45)*	24*–24.5 (0.08) and 29–31 (0.34)*
*LA 3*			
*a wave*	13–15.5 (n = 125)	13–15.5 (n = 135)	13–15.5 (n = 129)
	*13–13 (0.00) and 15–15.5 (0.20)*	*13–13.5 (0.20) and 15.5–16 (0.20)*	*12.5–13.5 (0.40) and 15.5–16.5 (0.40)*
*b wave*	27–31.5 (n = 125)	27–31.5 (n = 135)	27.5–32.5 (n = 129)
	*26.5–27 (0.11) and 30.5–32 (0.33)*	*26.5–27.5 (0.22) and 30.5–32 (0.33)*	*26.5–27.5 (0.20) and 32–33 (0.20)*

Timings are rounded to the nearest 0.5 ms. Numbers in italics represent the 90% confidence interval (CI) for each reference limit, followed in brackets by the 90% CI expressed as a proportion of the whole reference interval

**Table 5:** Age-specific nonparametric reference limits for amplitude of ISCEV-standard full-field ERGs with silver thread electrodes in the fornix position

Age	Nonparametric reference limit for ERG component amplitude (μV)		
	*90% CI of reference limits (CI as proportion of total reference interval)*		
	≤ 35	36–59	≥ 60
*DA 0.01*			
*b wave*	138–297 (n = 126)	115–279 (n = 130)	107–288 (n = 123)
	*130–142 (0.08) and 283–319 (0.23)*	*102–133 (0.19) and 266–315 (0.30)*	*100–119 (0.11) and 277–290 (0.07)*
*DA 3*			
*a wave*	119–238 (n = 130)	100–226 (n = 138)	80–220 (n = 130)
	*109–125 (0.13) and 227–261 (0.29)*	*90–105 (0.12) and 218–239 (0.17)*	*72–89 (0.12) and 201–234 (0.23)*
*b wave*	191–404 (n = 130)	167–377 (n = 138)	162–407 (n = 130)
	*178–203 (0.12) and 374–421 (0.22)*	*163–199 (0.17) and 361–469 (0.52)*	*151–171 (0. 08) and 377–458 (0.33)*
*DA 10*			
*a wave*	136–274 (n = 134)	124–277 (n = 136)	101–253 (n = 131)
	*130–148 (0.14) and 258–283 (0.18)*	*118–131 (0. 08) and 257–384 (0.18)*	*97–110 (0.09) and 235–280 (0.30)*
*b wave*	202–409 (n = 133)	191–387 (n = 132)	177–414 (n = 127)
	*192–208 (0.08) and 376–413 (0.18)*	*171–210 (0.20) and 379–481 (0.52)*	*170–187 (0.07) and 377–450 (0.31)*
*LA 30 Hz*			
*peak*	50–166 (n = 121)	45–140 (n = 137)	38–116 (n = 127)
	*41–52 (0.09) and 149–187 (0.33)*	*41–49 (0.09) and 118–175 (0.59)*	*36–42 (0.08) and 108–160 (0.66)*
*LA 3*			
*a wave*	12–43 (n = 125)	13–40 (n = 135)	12–35 (n = 129)
	*11–16 (0.17) and 39–49 (0.34)*	*8–15 (0.24) and 33–55 (0.83)*	*12–14 (0.11) and 31–41 (0.43)*
*b wave*	73–227 (n = 125)	64–172 (n = 135)	49–153 (n = 129)
	*54–77 (0.15) and 199–260 (0.40)*	*52–72 (0.19) and 152–247 (0.88)*	*45–59 (0.13) and 143–192 (0.46)*

Numbers in italics represent the 90% confidence interval (CI) for each reference limit, followed in brackets by the CI expressed as a proportion of the whole reference interval

**Fig. 4:**
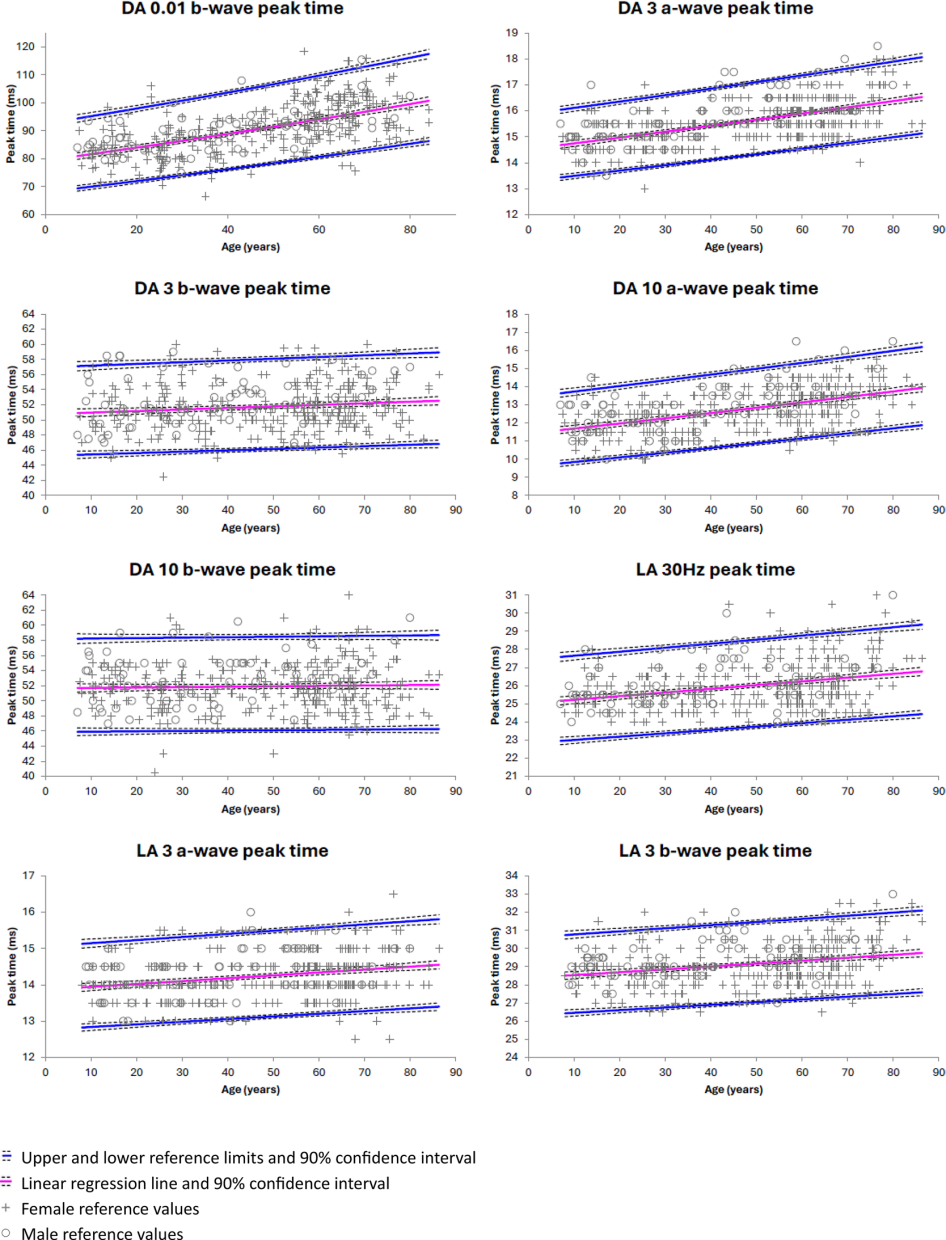
Age-adjusted parametric reference limits for peak time of the main DA 0.01, DA 3, DA 10, LA 30 Hz and LA 3 components. Calculated following Box-Cox transformation assuming homoscedasticity, using *Reference Values Adviser* for Excel [14]

**Fig. 5:**
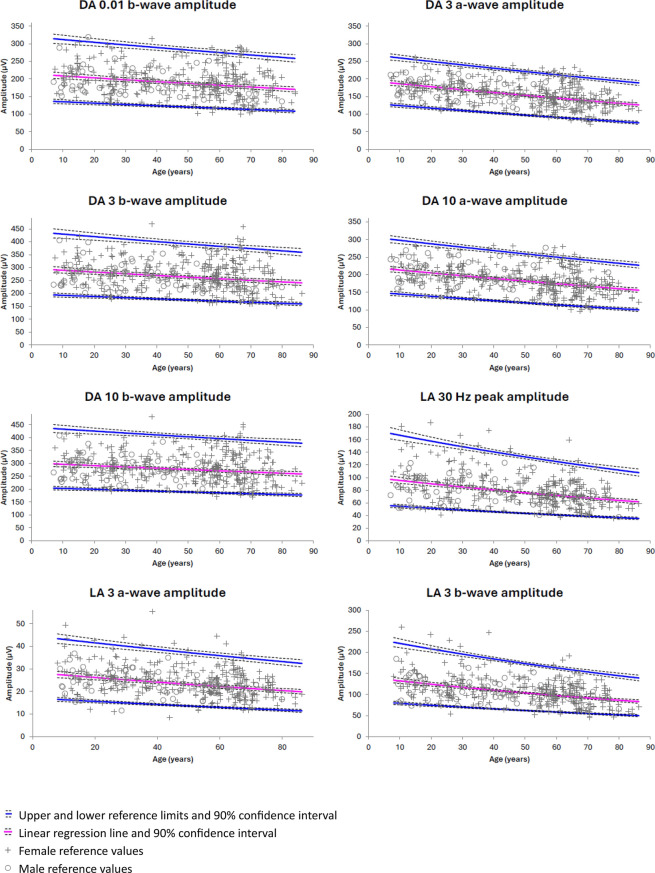
Age-adjusted reference limits for amplitude of the main DA 0.01, DA 3, DA 10, LA 30 Hz and LA 3 components. Calculated following Log_10_ transformation (LA 30 Hz and LA 3 b-wave only) or Box-Cox transformation (all other ERG component amplitudes), assuming homoscedasticity, using *Reference Values Adviser* for Excel [14]

The parametric age-adjusted reference limits, calculated following Log_10_ transformation (LA 30 Hz and LA 3 b-wave amplitude only) or Box-Cox transformation (all other ERG component amplitudes and peak times), are shown in Figs. 4 and 5 (also available in Online Resource 7). With this method, upper and lower reference limits had suitably narrow confidence intervals (≤ 20% of the reference interval) for all ERG component timings and amplitudes.

All eight ISCEV ERG components showed decreasing amplitude with age. The ERG component amplitudes most associated with age were the DA 3 and DA 10 a-waves (r^2^ = 0.20 and r^2^ = 0.16 respectively) and the LA 30 Hz and LA 3 responses (LA 30 Hz: r^2^ = 0.14, LA 3 a-wave: r^2^ = 0.10, LA 3 b-wave: r^2^ = 0.17). The dark-adapted b-wave components were least associated with age (DA 0.01 b-wave: r^2^ = 0.06, DA 3 b-wave: r^2^ = 0.05, DA 10 b-wave: r^2^ = 0.03). All but one ERG components showed increasing peak time with age. ERG component timings most correlated with age were DA 0.01 b-wave (r^2^ = 0.35), DA 3 a-wave (r^2^ = 0.32), and DA 10 a-wave (r^2^ = 0.25). DA 3 b-wave timing was poorly correlated with age (r^2^ = 0.02) and DA 10 b-wave timing showed no correlation with age (r^2^ < 0.01). LA 30 Hz, LA 3 a-wave, and LA 3 b-wave peak time had r^2^ of 0.11, 0.07, and 0.08 respectively.

## Discussion

This study demonstrates how, following validation, ERG reference data can be transferred between centres and electrode types to form a combined reference sample. The pooling of ISCEV Standard ERG reference data is justified by the high inter-centre consistency. This was shown in age-adjusted reference values which did not differ significantly between the two centres, and was confirmed in six subjects tested at both centres. The consistency of ISCEV standard ERGs across centres has previously been reported and likely results from the standardization of testing methods and recording parameters [3, 4]. The potential to transfer reference data between electrode types is also highlighted: there is a linear relationship between ERG amplitude recorded with gold foil and with silver thread electrodes, which justifies transference of reference data between these electrode types following suitable adjustment [5]. The combined sample containing 407 reference individuals was sufficiently large to allow calculation of age-adjusted reference limits across eight decades, which would not have been possible using any of the three samples alone.

CLSI guidelines outline several methods for calculation of reference limits [12]. Nonparametric methods may be preferable since they make no assumptions about the underlying distribution of the data. We included more than 120 reference subjects per age partition (≤ 35, 36–59, ≥ 60 years) to allow precise calculation of the 90% CI of the nonparametric reference limits. It is recommended that these confidence intervals should be small relative to the overall width of the reference interval (≤ 20% of total reference interval). This requirement was met for only some of the calculated reference limits, indicating that a larger sample would be required to meet this CLSI recommendation across all age groups, particularly for ERG peak times with narrow reference intervals such as DA 3, DA 10, and LA 3 a-wave peak time and LA 30 Hz peak time. Alternative methods include the use of parametric techniques following appropriate transformation. Box-Cox or Log_10_ transformation was applied to allow calculation of continuous parametric reference limits with age as a covariate. This method produced age-adjusted reference limits with suitably narrow 90% CIs for all ERG component timings and amplitudes. The age-adjusted ERG reference limits produced by both methods showed good consistency.

The reference data presented here demonstrate increasing ERG peak time and decreasing amplitude with age, in line with previous reports, highlighting the importance of age-adjusted reference values when interpreting ERG data [6, 7, 9]. The association with age varied between components. For dark-adapted responses, ERG a-wave amplitude was more closely associated with age than b-wave amplitude, accounting for 16–20% of the variation in DA 3 and DA 10 a-wave amplitude compared with less than 6% of the variation in DA 0.01, DA 3, and DA 10 b-wave amplitude. For light-adapted responses, 10–17% of the variation in amplitude could be explained by age. For peak time, DA 3 and DA 10 b-wave timing correlated poorly with age (r^2^ ≤ 0.02), while 25–35% of the variation in DA 0.01 b-wave, DA 3 a-wave, and DA 10 a-wave peak time could be explained by age. Approximately 7–11% of the variation in photopic ERG peak time could be explained by age. Although we employed a linear model to explore the relationship between ERG parameters and age, we note that the true relationship may be more complex. It is also noted that this is cross-sectional data and therefore does not necessarily reflect the change that would occur over an individual’s lifetime.

This study confirms the significant effect of electrode type on ERG amplitude, as reported in previously published studies [10, 15]. ERGs recorded with silver thread electrodes (fornix position) were approximately 55–65% of the amplitude obtained with gold foil electrodes, while ERGs recorded with skin electrodes were approximately 35% the amplitude of silver threads. ERG component peak times were consistent across electrode types, in line with previous reports [10]. Despite differences, reference intervals from one electrode type can be applied to patient data from another electrode type providing that a comparison analysis is carried out. It is highlighted that in many studies silver thread electrodes have been positioned on the lower eyelid rather than in the lower fornix, with ERG amplitudes reported to be 12–19% higher with no difference in peak time, and such data is likely amenable to a similar transformation [11, 16, 17].

To optimize diagnostic accuracy, patient ERGs should be interpreted against a reference range that closely matches the local patient population demographically [1, 5, 12]. This sample comprised mainly of adults (mean age 47 years), with relatively few paediatric cases, and given the rapid maturation of ERGs during early life [18–20] extrapolation to young children is precluded. The sample was 83% female, making partitioning of reference data by sex unfeasible, although data from both sexes appeared consistent when plotted against age. Other relevant partitioning factors such as ethnicity/pigmentation and refractive status were not captured. This is a limitation of the dataset, much of which was extracted retrospectively from clinical records and therefore lacked the details required when recruiting reference subjects prospectively (see [5]), including lack of a full medical examination to rule out any undiagnosed pathology. Given the potential variations in patient population, it is essential that centres wishing to adopt external reference data do so only after validation on local reference subjects, which can be achieved using the published methods ([5]) illustrated in this and other studies (e.g. [21]).

Patient ERGs should be interpreted against a reference range that was collected using closely comparable test methods [1, 5, 12]. Though the use of ISCEV Standard ERG stimuli promotes consistency across centres, there remains potential for variation in stimulus characteristics, partly because the ISCEV Standards tolerate some variation e.g., relating to light sources of stimuli and precise methods. Both centres in this study used the same LED-based ganzfeld stimulators to ensure consistency of stimulus characteristics, but other LED or xenon flashtube ganzfeld systems may produce light with differing properties that could alter ERG characteristics [1]. Of note, xenon flashtubes produce stimuli with a pulse width in the microsecond range, while the pulse width in the present study was 4 ms. Pulse width impacts peak time, particularly for the LA 30 Hz ERG and components with shorter peak times [22], further emphasizing the importance of local validation and, if necessary, application of a correction factor prior to clinical use. Although measurement of oscillatory potentials is an optional part of the ISCEV Standard full-field ERG analysis 1, these were not quantified in this study.

The reference datasets were collected with diverse sampling methods. Datasets 1 and 3 involved recruitment of volunteers without eye disease, while dataset 2 was an indirect sample drawn mainly from patients with normal test results, no detectable visual pathway pathology and a non-ophthalmic final diagnosis. Although direct sampling is seen as the gold standard, it can be challenging to recruit participants representative of the patient population in terms of ethnicity, socioeconomic background, education level, and other relevant demographics. Likewise, although indirect sampling draws directly from the patient population, eligible subjects may not be representative of the patient population as a whole. Males were under-represented across all samples, including the indirect sample which was mainly comprised of patients with non-organic visual loss that has a propensity to affect females more than males [23, 24]. Despite the differing sampling methods, the datasets did not differ significantly in amplitude or latency and combined to form a consistent reference sample. Dataset 3 consisted of twin pairs: when recalculated with half of each twin pair excluded, there was minimal change in the reference limits.

## Conclusion

ISCEV Standard ERG reference data can be transferred between centres, justified by the high inter-centre consistency of responses. Reference data may also be transferred following a change in test methods, such as corneal electrode type, subject to comparison analysis and suitable adjustment. The pooled data forms a larger, more robust reference range which allows for more precise calculation of age-adjusted reference limits, pertinent to clinical management, standardization, and multi-centre studies.

## Supplementary Information

Below is the link to the electronic supplementary material.

Supplementary file1 (PDF 165 kb)

Supplementary file2 (PDF 221 kb)

Supplementary file3 (PDF 162 kb)

Supplementary file4 (PDF 160 kb)

Supplementary file5 (PDF 159 kb)

Supplementary file6 (PDF 231 kb)

Supplementary file7 (XLSX 483 kb)
