# Supplementary material for: ISCEV standard full-field ERG reference limits from 407 healthy subjects, derived from transference and validation of reference data between electrode types and centres
Source: Doc Ophthalmol. 2025 Apr 1;150(2):47–64. doi: 10.1007/s10633-025-10009-2 (PMC11991937; doi:10.1007/s10633-025-10009-2)
Supplement: Supplementary file 1 — Supplementary file1 (PDF 165 kb) [file 10633_2025_10009_MOESM1_ESM.pdf]

Supplementary Information: Online Resource 1

(i) General settings

	Centre 1 (MEH)	Centre 2 (STH)
**Electrode setup**		
**Active electrode type and position**	Silver thread electrode in the lower fornix	Silver thread electrode in the lower fornix
**Reference electrode type and position**	Skin electrode, ipsilateral outer canthus *(10-mm reusable EEG Ag/Au electrodes, MFI-BV)*	Skin electrode, ipsilateral outer canthus *(24-mm disposable ground electrode, Unimed Electrode Supplies Limited)*
**Ground electrode type and position**	Skin electrode, forehead *(10-mm reusable EEG Ag/Au electrodes, MFI-BV)*	Skin electrode, forehead *(24-mm disposable ground electrode, Unimed Electrode Supplies Limited)*
**Amplifier settings**		
**Low frequency cut-off**	0.312 Hz	0.312 Hz
**High frequency cut-off**	500 Hz	300 Hz
**Notch filter used?**	No	No
**Sample rate**	2 kHz	2 kHz
**Light characteristics**		
**Duration of dark adaptation**	20 minutes	20 minutes
**Duration of light adaptation**	10 minutes	10 minutes
**Stimulus type**	LED, white light at 6500K	LED, white light at 6500K
**Pulse width**	4 ms	Up to 4 ms

“ISCEV standard full-field ERG reference limits from 407 healthy subjects, derived from transference and validation of reference data between electrode types and centres.” *Documenta Ophthalmologica*. RA Baker[1], SM Leo[1,2], WI Clowes[1], I Chow[3], X Jiang[2,3], AL Georgiou[1,2], A Calcagni[1], CJ Hammond[3], MM Neveu[1,2], OA Mahroo[1,2,3], AG Robson[1,2]. Affiliations: 1. Moorfields Eye Hospital NHS Foundation Trust. 2. UCL Institute of Ophthalmology, London. 3. St Thomas’ Hospital, London. Corresponding author e-mail: anthony.robson3@nhs.net

(ii) Step settings

	Centre 1 (MEH)	Centre 2 (STH)
**DA 0.01**		
**Time-integrated luminance**	0.01 photopic $cd{\cdot}s{\cdot}m^{-2}$	0.01 photopic $cd{\cdot}s{\cdot}m^{-2}$
**Inter-stimulus interval**	≥2 s	≥5 s
**Pre-stimulus delay**	10 ms	20 ms
**DA 3**		
**Time-integrated luminance**	3.0 photopic $cd{\cdot}s{\cdot}m^{-2}$	3.0 photopic $cd{\cdot}s{\cdot}m^{-2}$
**Inter-stimulus interval**	≥10 s	≥20 s
**Pre-stimulus delay**	20 ms	20 ms
**DA 10**		
**Time-integrated luminance**	10.0 photopic $cd{\cdot}s{\cdot}m^{-2}$	10.0 photopic $cd{\cdot}s{\cdot}m^{-2}$
**Inter-stimulus interval**	≥20 s	≥20 s
**Pre-stimulus delay**	20 ms	20 ms
**LA 30 Hz**		
**Time-integrated luminance**	3.0 photopic $cd{\cdot}s{\cdot}m^{-2}$	3.0 photopic $cd{\cdot}s{\cdot}m^{-2}$
**Inter-stimulus interval**	0 s	0 s
**Pre-stimulus delay**	0 ms	0 ms
**LA 3**		
**Time-integrated luminance**	3.0 photopic $cd{\cdot}s{\cdot}m^{-2}$	3.0 photopic $cd{\cdot}s{\cdot}m^{-2}$
**Inter-stimulus interval**	0.5 s	0.5 s
**Pre-stimulus delay**	20 ms	20 ms

"ISCEV standard full-field ERG reference limits from 407 healthy subjects, derived from transference and validation of reference data between electrode types and centres." *Documenta Ophthalmologica*. RA Baker[1], SM Leo[1,2], WI Clowes[1], I Chow[3], X Jiang[2,3], AL Georgiou[1,2], A Calcagni[1], CJ Hammond[3], MM Neveu[1,2], OA Mahroo[1,2,3], AG Robson[1,2]. Affiliations: 1. Moorfields Eye Hospital NHS Foundation Trust. 2. UCL Institute of Ophthalmology, London. 3. St Thomas' Hospital, London. Corresponding author e-mail: anthony.robson3@nhs.net
