# Supplementary material for: ISCEV standard full-field ERG reference limits from 407 healthy subjects, derived from transference and validation of reference data between electrode types and centres
Source: Doc Ophthalmol. 2025 Apr 1;150(2):47–64. doi: 10.1007/s10633-025-10009-2 (PMC11991937; doi:10.1007/s10633-025-10009-2)
Supplement: Supplementary file 2 — Supplementary file2 (PDF 221 kb) [file 10633_2025_10009_MOESM2_ESM.pdf]

Supplementary information: Online resource 2

(i) Comparison of ERGs recorded with two types of silver thread electrode

Full-field ERGs (ganzfeld stimulation, Diagnosys ColorDome) were recorded in 4 healthy adult subjects using a Sterile ERG Thread Electrode from Spesmedica in the right eye and a DTL Plus electrode from Diagnosys in the left eye, each referred to a skin electrode at the ipsilateral outer canthus with the ground electrode placed on the forehead. Mydriasis was applied with pupil diameter being ≥7mm and equal in both eyes. The stimuli involved a stimulus-response series of 11 flash strengths (white light, 6500K) ranging from 0.001 cd.s.m^{-2} to 30.0 cd.s.m^{-2} under dark-adapted conditions (20 minutes' dark adaptation), incorporating the ISCEV standard steps (DA 0.01, DA 3, DA 10). The participants were then light adapted for 10 minutes and underwent photopic 30Hz flicker (LA 30 Hz) and single flash (LA 3) ERG testing. For each subject, the amplitude and peak time of the response from each electrode (i.e. from each eye) for each stimulus setting was plotted to find the r^2 value, slope, and intercept of the line (see figure A for an example). Recordings were highly consistent between the two electrodes, having an approximately 1:1 relationship as demonstrated in Table A.

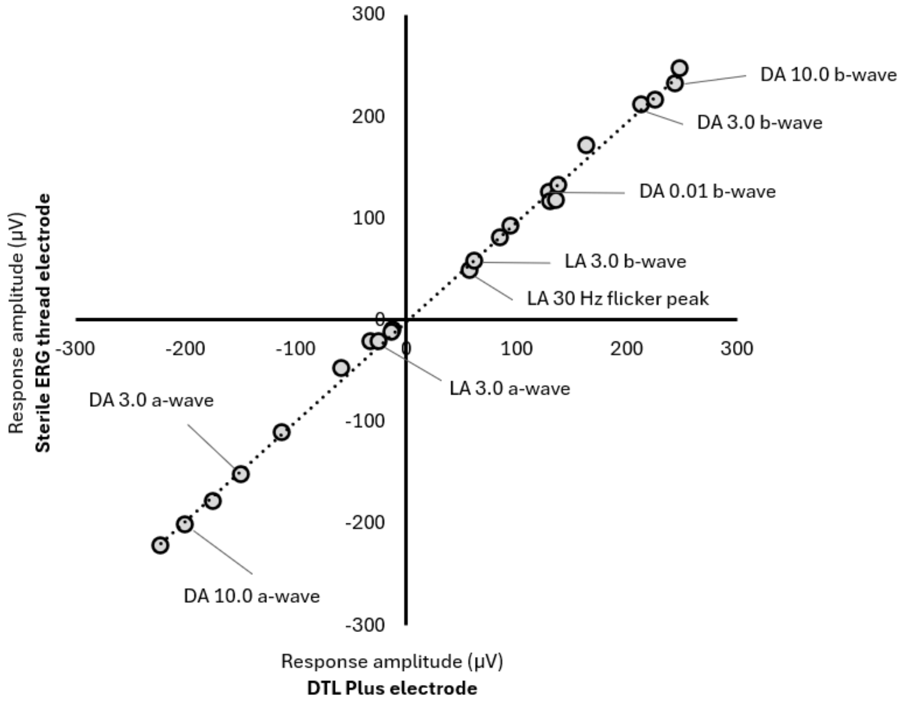

Figure A: A comparison of ERG response amplitude recorded simultaneously with two types of silver thread electrode across 13 recording conditions in Subject 3. (Sterile ERG thread electrode from Spesmedica: right eye; DTL Plus electrode from Diagnosys: left eye.) Dashed line shows simple linear regression. Unlabelled data points relate to intermediate flash strengths.

	Response amplitude			Response peak time		
	r^2	slope	intercept	r^2	slope	intercept
Subject 1	0.99	0.90	-2.3	0.99	0.95	2.0
Subject 2	>0.99	0.91	-1.2	>0.99	0.98	0.4
Subject 3	>0.99	0.98	-1.7	>0.99	0.95	1.3
Subject 4	0.99	0.99	-5.0	>0.99	0.98	0.7

Table A: Linear regression of ERG response amplitude and peak time in four subjects tested with two types of silver thread electrode across 13 recording conditions. (Sterile ERG thread electrode from Spesmedica: right eye; DTL Plus electrode from Diagnosys: left eye.)

"ISCEV standard full-field ERG reference limits from 407 healthy subjects, derived from transference and validation of reference data between electrode types and centres." *Documenta Ophthalmologica*. RA Baker[1], SM Leo[1,2], WIN Clowes[1], I Chow[3], X Jiang[2,3], AL Georgiou[1,2], A Calcagni[1], CJ Hammond[3], MM Neveu[1,2], OA Mahroo[1,2,3], AG Robson[1,2]. Affiliations: 1. Moorfields Eye Hospital NHS Foundation Trust. 2. UCL Institute of Ophthalmology, London. 3. St Thomas' Hospital, London. Corresponding author e-mail: anthony.robson3@nhs.net

(ii) Validation of multi-electrode ERG setup used to compare active electrode types.

Subjects 1-4 formed part of 'Dataset 1' and as such were additionally tested with the simultaneous gold foil / silver thread / skin active electrode montage described in *Methods: Datasets* (Dataset 1b), with both tests performed within a 2-month period. As the latter recordings involved simultaneous reference of a silver thread electrode and a skin electrode both applied to the same eye and referenced to the same reference electrode, additional validation was performed to assure that the reference of two active electrodes to a single reference electrode did not alter ERG response characteristics. Left eye ISCEV Standard responses recorded in the standard 'single active to single reference electrode' montage described in section (i) and the non-standard 'two actives to single reference electrode' montage described in *Methods: Datasets* were compared. In all instances the active electrode used was the DTL Plus electrode (Diagnosys). The amplitude and peak time of ISCEV Standard responses recorded with both electrode montages were plotted to find the r^2 value, slope, and intercept of the line for each participant (see Figure B for an example). Recordings were highly consistent between the two recording montages, having an approximately 1:1 relationship as demonstrated in table B.

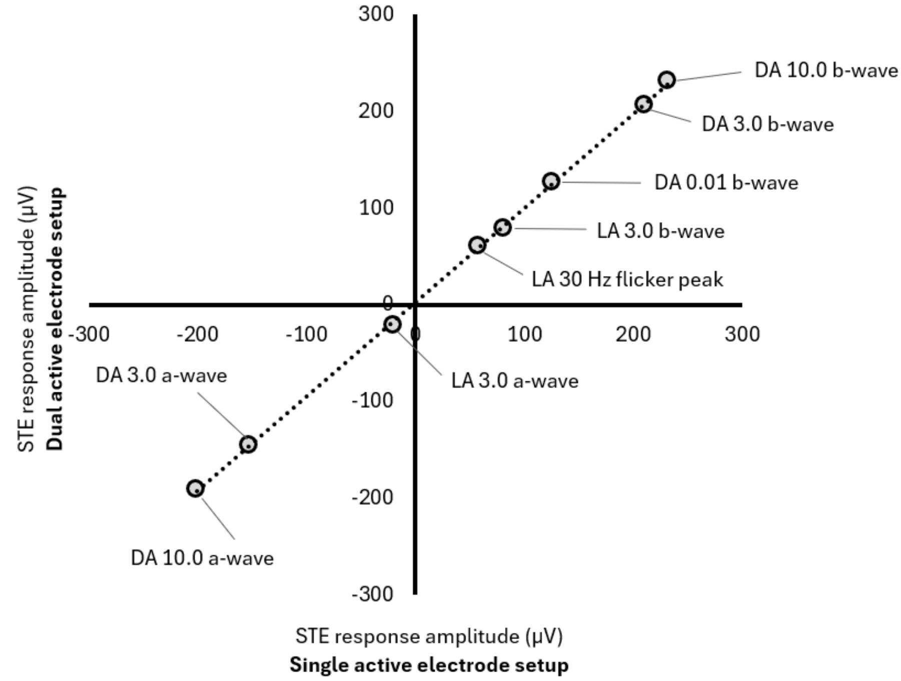

Figure B: A comparison of ERG response amplitude recorded from the left eye of Subject 3 using a DTL Plus electrode with two electrode montages: standard 'single active electrode to single reference electrode', and non-standard 'two active electrodes to single reference electrode'. Dashed line shows simple linear regression.

	Response amplitude			Response peak time		
	r^2	slope	intercept	r^2	slope	intercept
Subject 1	>0.99	1.00	-1.9	>0.99	1.01	-0.4
Subject 2	>0.99	0.96	-1.7	>0.99	0.99	0.1
Subject 3	>0.99	0.97	2.9	>0.99	0.98	0.2
Subject 4	>0.99	0.95	-1.2	>0.99	0.99	-0.2

Table B: Linear regression of ERG response amplitude and peak time recorded in 'single active to single reference electrode' montage versus 'two actives to single reference electrode' montage in four participants (see text for details).

"ISCEV standard full-field ERG reference limits from 407 healthy subjects, derived from transference and validation of reference data between electrode types and centres." *Documenta Ophthalmologica*. RA Baker[1], SM Leo[1,2], WIN Clowes[1], I Chow[3], X Jiang[2,3], AL Georgiou[1,2], A Calcagni[1], CJ Hammond[3], MM Neveu[1,2], OA Mahroo[1,2,3], AG Robson[1,2]. Affiliations: 1. Moorfields Eye Hospital NHS Foundation Trust. 2. UCL Institute of Ophthalmology, London. 3. St Thomas' Hospital, London. Corresponding author e-mail: anthony.robson3@nhs.net
