# Supplementary material for: ISCEV standard full-field ERG reference limits from 407 healthy subjects, derived from transference and validation of reference data between electrode types and centres
Source: Doc Ophthalmol. 2025 Apr 1;150(2):47–64. doi: 10.1007/s10633-025-10009-2 (PMC11991937; doi:10.1007/s10633-025-10009-2)
Supplement: Supplementary file 3 — Supplementary file3 (PDF 162 kb) [file 10633_2025_10009_MOESM3_ESM.pdf]

Supplementary Information: Online Resource 3

	n=	Bland-Altman Bias<br>*95% LOA*<br>Amplitude (Skin:STE)	Peak time (Skin - STE, ms)
DA 0.01 b-wave	33	0.35<br>*0.22 – 0.49*	-1.9<br>*-10.5 – 6.6*
DA 3 a-wave	35	0.37<br>*0.23 – 0.50*	-0.1<br>*-0.8 – 0.6*
DA 3 b-wave	35	0.35<br>*0.22 – 0.48*	-2.0<br>*-10.6 – 6.6*
DA 10 a-wave	36	0.36<br>*0.23 – 0.48*	-0.4<br>*-1.9 – 1.1*
DA 10 b-wave	36	0.35<br>*0.22 – 0.47*	-2.9<br>*-14 – 8.2*
LA 30 Hz peak	38	0.38<br>*0.26 – 0.50*	-0.2<br>*-0.8 – 0.4*
LA 3 a-wave	38	0.36<br>*0.18 – 0.54*	-0.4<br>*-2.1 – 1.3*
LA 3 b-wave	38	0.36<br>*0.23 – 0.50*	-0.2<br>*-1.0 – 0.5*

Table shows Bland-Altman analysis of skin electrode ERGs recorded simultaneously with silver thread ERGs from the left eye.

"ISCEV standard full-field ERG reference limits from 407 healthy subjects, derived from transference and validation of reference data between electrode types and centres." *Documenta Ophthalmologica*. RA Baker[1], SM Leo[1,2], WIN Clowes[1], I Chow[3], X Jiang[2,3], AL Georgiou[1,2], A Calcagni[1], CJ Hammond[3], MM Neveu[1,2], OA Mahroo[1,2,3], AG Robson[1,2]. Affiliations: 1. Moorfields Eye Hospital NHS Foundation Trust. 2. UCL Institute of Ophthalmology, London. 3. St Thomas' Hospital, London. Corresponding author e-mail: anthony.robson3@nhs.net
