# Supplementary material for: ISCEV standard full-field ERG reference limits from 407 healthy subjects, derived from transference and validation of reference data between electrode types and centres
Source: Doc Ophthalmol. 2025 Apr 1;150(2):47–64. doi: 10.1007/s10633-025-10009-2 (PMC11991937; doi:10.1007/s10633-025-10009-2)
Supplement: Supplementary file 4 — Supplementary file4 (PDF 160 kb) [file 10633_2025_10009_MOESM4_ESM.pdf]

Supplementary Information: Online Resource 4

		Bland-Altman Bias<br>*95% LOA*	
	n=	Amplitude<br>(Skin:GFE)	Peak time<br>(Skin - GFE, ms)
DA 0.01 b-wave	39	0.19<br>*0.11 – 0.27*	-3.8<br>*-13 – 5.1*
DA 3 a-wave	41	0.21<br>*0.12 – 0.29*	-0.3<br>*-1.9 – 1.0*
DA 3 b-wave	41	0.19<br>*0.11 – 0.27*	-3.2<br>*-11 – 4.6*
DA 10 a-wave	41	0.20<br>*0.12 – 0.29*	-0.9<br>*-2.5 – 0.6*
DA 10 b-wave	41	0.19<br>*0.10 – 0.28*	0.9<br>*0.8 – 1.1*
LA 30 Hz peak	41	0.24<br>*0.14 – 0.34*	-0.3<br>*-1.0 – 0.4*
LA 3 a-wave	41	0.20<br>*0.11 – 0.29*	-0.8<br>*-3.1 – 1.5*
LA 3 b-wave	41	0.23<br>*0.13 – 0.32*	-0.5<br>*-1.3 – 0.3*

Table shows Bland-Altman analysis of skin electrode ERGs recorded simultaneously with gold foil ERGs from the right eye

"ISCEV standard full-field ERG reference limits from 407 healthy subjects, derived from transference and validation of reference data between electrode types and centres." *Documenta Ophthalmologica*. RA Baker[1], SM Leo[1,2], WIN Clowes[1], I Chow[3], X Jiang[2,3], AL Georgiou[1,2], A Calcagni[1], CJ Hammond[3], MM Neveu[1,2], OA Mahroo[1,2,3], AG Robson[1,2]. Affiliations: 1. Moorfields Eye Hospital NHS Foundation Trust. 2. UCL Institute of Ophthalmology, London. 3. St Thomas' Hospital, London. Corresponding author e-mail: anthony.robson3@nhs.net
