# Supplementary material for: ISCEV standard full-field ERG reference limits from 407 healthy subjects, derived from transference and validation of reference data between electrode types and centres
Source: Doc Ophthalmol. 2025 Apr 1;150(2):47–64. doi: 10.1007/s10633-025-10009-2 (PMC11991937; doi:10.1007/s10633-025-10009-2)
Supplement: Supplementary file 5 — Supplementary file5 (PDF 159 kb) [file 10633_2025_10009_MOESM5_ESM.pdf]

Supplementary Information: Online Resource 5

	n=	Bland-Altman Bias<br>*95% LOA*<br>Amplitude (RE:LE)	Peak time (LE - RE, ms)
DA 0.01 b-wave	39	1.00<br>*0.71 – 1.29*	-0.5<br>*-3.5 – 2.6*
DA 3 a-wave	41	1.03<br>*0.72 – 1.34*	-0.1<br>*-1.6 – 1.4*
DA 3 b-wave	41	1.00<br>*0.73 – 1.27*	-0.1<br>*-5.8 – 5.6*
DA 10 a-wave	41	1.01<br>*0.75 – 1.27*	-0.1<br>*-2.4 – 2.3*
DA 10 b-wave	41	0.99<br>*0.75 – 1.23*	-0.9<br>*-8.5 – 6.8*
LA 30 Hz peak	41	1.01<br>*0.69 – 1.32*	0.0<br>*-0.8 – 0.8*
LA 3 a-wave	41	1.04<br>*0.60 – 1.48*	0.3<br>*-2.4 – 3.0*
LA 3 b-wave	41	0.99<br>*0.75 – 1.24*	-0.1<br>*-0.7 – 0.6*

Table shows Bland-Altman analysis of skin electrode ERGs recorded simultaneously from right and left eyes

"ISCEV standard full-field ERG reference limits from 407 healthy subjects, derived from transference and validation of reference data between electrode types and centres." *Documenta Ophthalmologica*. RA Baker[1], SM Leo[1,2], WIN Clowes[1], I Chow[3], X Jiang[2,3], AL Georgiou[1,2], A Calcagni[1], CJ Hammond[3], MM Neveu[1,2], OA Mahroo[1,2,3], AG Robson[1,2]. Affiliations: 1. Moorfields Eye Hospital NHS Foundation Trust. 2. UCL Institute of Ophthalmology, London. 3. St Thomas' Hospital, London. Corresponding author e-mail: anthony.robson3@nhs.net
