# Supplementary material for: ISCEV standard full-field ERG reference limits from 407 healthy subjects, derived from transference and validation of reference data between electrode types and centres
Source: Doc Ophthalmol. 2025 Apr 1;150(2):47–64. doi: 10.1007/s10633-025-10009-2 (PMC11991937; doi:10.1007/s10633-025-10009-2)
Supplement: Supplementary file 6 — Supplementary file6 (PDF 231 kb) [file 10633_2025_10009_MOESM6_ESM.pdf]

Supplementary Information: Online Resource 6

	Nonparametric reference limit for ERG component amplitude (µV)<br>*90% CI of reference limits (CI as proportion of total reference interval)*		
**Age**	**≤35**	**36 - 59**	**≥60**
DA 0.01<br>***b wave***	140 – 297 (n=121)<br>*130 – 148 (0.11) and 283 – 319 (0.23)*	116 – 268 (n=100)<br>*102 – 132 (0.20) and 252 – 315 (0.41)*	112 – 284 (n=66)<br>*107 – 123 (0.09) and 270 – 289 (0.11)*
DA 3<br>***a wave***	123 – 239 (n=125)<br>*109 – 130 (0.18) and 227 – 261 (0.30)*	100 – 229 (n=105)<br>*98 – 105 (0.06) and 220 – 239 (0.15)*	82 – 211 (n=72)<br>*72 – 92 (0.15) and 195 – 214 (0.15)*
b wave	190 – 405 (n=125)<br>*178 – 203 (0.12) and 374 – 421 (0.22)*	168 – 381 (n-105)<br>*163 – 196 (0.16) and 362 – 469 (0.50)*	170 – 402 (n=71)<br>*167 – 176 (0.04) and 364 – 409 (0.19)*
DA 10<br>***a wave***	146 – 274 (n=129)<br>*137 – 154 (0.14) and 262 – 283 (0.16)*	125 - 279 (n=105)<br>*121 – 130 (0.05) and 265 – 384 (0.12)*	101 – 252 (n=72)<br>*101 – 111 (0.07) and 235 – 280 (0.30)*
b wave	202 – 409 (n=129)<br>*192 – 212 (0.09) and 379 – 413 (0.17)*	192 – 389 (n=103)<br>*171 – 207 (0.18) and 382 – 481 (0.50)*	177 – 422 (n=71)<br>*170 – 192 (0.09) and 371 – 450 (0.33)*
LA 30 Hz<br>***peak***	50 – 168 (n=116)<br>*41 – 52 (0.09) and 150 – 187 (0.32)*	43 – 143 (n=105)<br>*41 – 48 (0.07) and 121 – 175 (0.53)*	37 – 134 (n=70)<br>*36 – 40 (0.05) and 110 – 160 (0.51)*
LA 3<br>***a wave***	12 – 43 (n=120)<br>*11 – 16 (0.17) and 39 – 49 (0.34)*	12 – 39 (n=103)<br>*8 – 15 (0.24) and 33 – 55 (0.81)*	12 – 38 (n=71)<br>*12 – 15 (0.12) and 32 – 41 (0.37)*
b wave	73 – 228 (n=120)<br>*54 – 79 (0.16) and 199 – 260 (0.39)*	64 – 175 (n=103)<br>*52 – 71 (0.17) and 154 – 247 (0.83)*	47 – 184 (n=71)<br>*45 – 57 (0.09) and 143 – 192 (0.35)*

(i) Age-specific nonparametric reference limits for amplitude of ISCEV-standard full-field ERGs with silver thread electrodes in the fornix position with paired (twin) data excluded. Where n<120, the 90% CI of the reference limits was calculated using a bootstrap method.

"ISCEV standard full-field ERG reference limits from 407 healthy subjects, derived from transference and validation of reference data between electrode types and centres." *Documenta Ophthalmologica*. RA Baker[1], SM Leo[1,2], WIN Clowes[1], I Chow[3], X Jiang[2,3], AL Georgiou[1,2], A Calcagni[1], CJ Hammond[3], MM Neveu[1,2], OA Mahroo[1,2,3], AG Robson[1,2]. Affiliations: 1. Moorfields Eye Hospital NHS Foundation Trust. 2. UCL Institute of Ophthalmology, London. 3. St Thomas' Hospital, London. Corresponding author e-mail: anthony.robson3@nhs.net

Supplementary Information: Online Resource 6

	**Nonparametric reference limits for ERG component timing (ms)**<br>*90% CI of reference limits (CI as proportion of total reference interval)*		
**Age**	**≤35**	**36 - 59**	**≥60**
DA 0.01<br>***b wave***	71.5 – 98.5 (n=121)<br>*66.5 – 75.5 (0.34) and 94.5 – 106 (0.43)*	76 – 106.5 (n=100)<br>73 – 79.5 (0.21) and 104 – 108 (0.12)	77.5 – 115.5 (n=66)<br>*77.5 – 86 (0.22) and 110.5 – 116 (0.15)*
DA 3<br>***a wave***<br><br>***b wave***	13.5 – 16 (n=125)<br>*13 – 14 (0.41) and 16 – 17 (0.41)*<br><br>45.5 – 58.5 (n=125)<br>*42.5 – 47 (0.35) and 57.5 – 60 (0.19)*	14.5 – 17 (n=105)<br>*14.5 – 15 (0.12) and 17 – 17.5 (0.19)*<br><br>46.5 – 59.5 (n=105)<br>*45 – 46.5 (0.11) and 57 – 59.5 (0.18)*	14.5 – 18 (n=72)<br>*14 – 15 (0.25) and 17.5 – 18.5 (0.27)*<br><br>47 – 59 (n=71)<br>*45.5 – 48.5 (0.23) and 57.5 – 60 (0.20)*
DA 10<br>***a wave***<br><br>***b wave***	10 – 14.5 (n=129)<br>*10 – 10.5 (0.11) and 13.5 – 14.5 (0.23)*<br><br>47 – 59.5 (n=303)<br>*43 – 47.5 (0.36) and 59 – 61 (0.16)*	11 – 15 (n=105)<br>*11 – 11.5 (0.08) and 14.5 – 16.5 (0.50)*<br><br>*b-wave: see ≤35 years*	11.5 – 15.5 (n=72)<br>*11 – 11.5 (0.12) and 15 – 16.5 (0.33)*<br><br>*b-wave: see ≤35 years*
LA 30 Hz<br>***peak***	24 – 28 (n=116)<br>*24 – 24.5 (0.12) and 27 – 28 (0.24)*	24 – 30 (n=105)<br>*24 – 24.5 (0.05) and 28 – 30.5 (0.39)*	24 – 30.5 (n=70)<br>*24 – 24.5 (0.08) and 29 – 31 (0.30)*
LA 3<br>***a wave***<br><br>***b wave***	13 – 15.5 (n=120)<br>*13 – 13 (0.00) and 15.0 – 15.5 (0.20)*<br><br>27 – 31.5 (n=120)<br>*26.5 – 27 (0.11) and 30.5 – 32 (0.33)*	13 – 15.5 (n=103)<br>*13 – 13.5 (0.20) and 15.5 – 16 (0.20)*<br><br>27 – 31.5 (n=103)<br>*26.5 – 27.5 (0.21) and 31 – 32 (0.21)*	12.5 – 15.5 (n=71)<br>*12.5 – 13.5 (0.29) and 15.5 – 16 (0.16)*<br><br>27.5 – 32.5 (n=71)<br>*26.5 – 27.5 (0.23) and 31.5 – 33 (0.25)*

(ii) Age-specific nonparametric reference limits for timing of ISCEV-standard full-field ERGs with silver thread electrodes in the fornix position, with paired (twin) data excluded. Timings are rounded to the nearest 0.5ms. Where n<120, the 90% CI of the reference limits was calculated using a bootstrap method.

"ISCEV standard full-field ERG reference limits from 407 healthy subjects, derived from transference and validation of reference data between electrode types and centres." *Documenta Ophthalmologica*. RA Baker[1], SM Leo[1,2], WIN Clowes[1], I Chow[3], X Jiang[2,3], AL Georgiou[1,2], A Calcagni[1], CJ Hammond[3], MM Neveu[1,2], OA Mahroo[1,2,3], AG Robson[1,2]. Affiliations: 1. Moorfields Eye Hospital NHS Foundation Trust. 2. UCL Institute of Ophthalmology, London. 3. St Thomas' Hospital, London. Corresponding author e-mail: anthony.robson3@nhs.net
